# OSU-2S/Sorafenib Synergistic Antitumor Combination against Hepatocellular Carcinoma: The Role of PKCδ/p53

**DOI:** 10.3389/fphar.2016.00463

**Published:** 2016-11-30

**Authors:** Hany A. Omar, Mai F. Tolba, Jui-Hsiang Hung, Taleb H. Al-Tel

**Affiliations:** ^1^Sharjah Institute for Medical Research and College of Pharmacy, University of SharjahSharjah, United Arab Emirates; ^2^Department of Pharmacology and Toxicology, Faculty of Pharmacy, Beni-Suef UniversityBeni-Suef, Egypt; ^3^Department of Pharmacology and Toxicology, Faculty of Pharmacy, Ain Shams UniversityCairo, Egypt; ^4^School of Pharmacy, Chapman University, IrvineCA, USA; ^5^Department of Biotechnology, Chia Nan University of Pharmacy and ScienceTainan, Taiwan

**Keywords:** OSU-2S, sorafenib, hepatocellular carcinoma, cancer resistance, PKCδ, p53

## Abstract

**Background:** Sorafenib (Nexavar^®^) is an FDA-approved systemic therapy for advanced hepatocellular carcinoma (HCC). However, the low efficacy and adverse effects at high doses limit the clinical application of sorafenib and strongly recommend its combination with other agents aiming at ameliorating its drawbacks. OSU-2S, a PKCδ activator, was selected as a potential candidate anticancer agent to be combined with sorafenib to promote the anti-cancer activity through synergistic interaction.

**Methods:** The antitumor effects of sorafenib, OSU-2S and their combination were assessed by MTT assay, caspase activation, Western blotting, migration/invasion assays in four different HCC cell lines. The synergistic interactions were determined by Calcusyn analysis. PKCδ knockdown was used to elucidate the role of PKCδ activation as a mechanism for the synergy. The knockdown/over-expression of p53 was used to explain the differential sensitivity of HCC cell lines to sorafenib and/or OSU-2S.

**Results:** OSU-2S synergistically enhanced the anti-proliferative effects of sorafenib in the four used HCC cell lines with combination indices <1. This effect was accompanied by parallel increases in caspase 3/7 activity, PARP cleavage, PKCδ activation and inhibition of HCC cell migration/invasion. In addition, PKCδ knockdown abolished the synergy between sorafenib and OSU-2S. Furthermore, p53 restoration in Hep3B cells through the over-expression rendered them more sensitive to both agents while p53 knockdown from HepG2 cells increased their resistance to both agents.

**Conclusion:** OSU-2S augments the anti-proliferative effect of sorafenib in HCC cell lines, in part, through the activation of PKCδ. The p53 status in HCC cells predicts their sensitivity toward both sorafenib and OSU-2S. The proposed combination represents a therapeutically relevant approach that can lead to a new HCC therapeutic protocol.

## Introduction

Hepatocellular carcinoma (HCC) is the most common type of liver tumors and a leading cause of cancer-associated death worldwide. HCC usually develops as a primary malignancy in patients suffering from chronic liver diseases and liver cirrhosis (Omar et al., 2011). A major challenge in the non-operative management of HCC is the cellular resistance to conventional anticancer agents, which may be attributed to the heterogeneity of genetic abnormalities acquired during the course of carcinogenesis (Pancione et al., 2012).

Sorafenib is an orally bioavailable multikinase inhibitor, which is approved for the treatment of unresectable advanced HCC (Wilhelm et al., 2006; Llovet et al., 2008). It works mainly through the inhibition of cancer cell survival pathways, such as RAF kinases, vascular endothelial growth factor and platelet-derived growth factor (Strumberg, 2005). Among many other targeted therapies for HCC, which are under development, sorafenib is currently the only FDA-approved systemic therapy for advanced HCC (Worns and Galle, 2010). However, in clinical practice, sorafenib exhibited low efficacy with a limited improvement in the median survival of HCC patients, which could be due to *de novo* resistance or the dose reductions to avoid the full dose adverse effects (Al-Rajabi et al., 2015; Federico et al., 2015). Therefore, combination therapies with sorafenib aiming at increasing the anticancer efficacy and reducing the required doses and consequently, minimizing the adverse effects and prolonging the patient survival are strongly encouraged (Hikita et al., 2010; Xie et al., 2012; Hu et al., 2016). In addition, the need for combination therapy is supported by the fact that targeting cell survival pathways in cancer cells by monotherapy is usually unsuccessful due to the ability of cancer cells to compensate for the affected targets by activating alternative compensatory pathway, a phenomenon known as redundancy (Li et al., 2014; Lavi, 2015).

One of the successful approaches in combination therapy is to select novel agents targeting different signaling pathways without significant systemic toxicity (Morisaki et al., 2013). Accordingly, OSU-2S was selected as a potential candidate anticancer agent to be combined with sorafenib to promote the anti-cancer activity and lower their therapeutic doses through the possible synergistic efficacy. OSU-2S is a novel anti-cancer agent that was designed and developed to selectively avert the immunosuppressive effects and related toxicities of its predecessor analog, FTY720 (Adachi and Chiba, 2008; Omar et al., 2011; Mao et al., 2014).

Previous *in vitro* studies showed the promising cytotoxicity of OSU-2S in many cancer cells, such as chronic lymphocytic leukemia (CLL), mantle cell lymphoma (MCL), acute lymphoblastic leukemia (ALL) (Bai et al., 2011). OSU-2S also demonstrated high efficiency in suppressing HCC *in vivo* without causing any immunosuppressive effect (Omar et al., 2011). The anti-proliferative mechanism of OSU-2S in HCC is mediated through the activation of reactive oxygen species-PKCδ signaling pathways and the subsequent induction of caspase-dependent apoptosis (Omar et al., 2011).

In the current study, we aimed to test the potential synergy between OSU-2S and sorafenib as a new therapeutic modality for the treatment of HCC which can exploit the maximal benefit through mechanistic synergy. We hypothesize that OSU-2S-induced modulation of PKCδ/p53 signaling plays a key role in augmenting sorafenib antitumor activity in HCC cells. The suggested combination therapy should increase sorafenib therapeutic gain and address the recently expressed safety concerns.

## Materials and Methods

### Material

**FIGURE 1:**
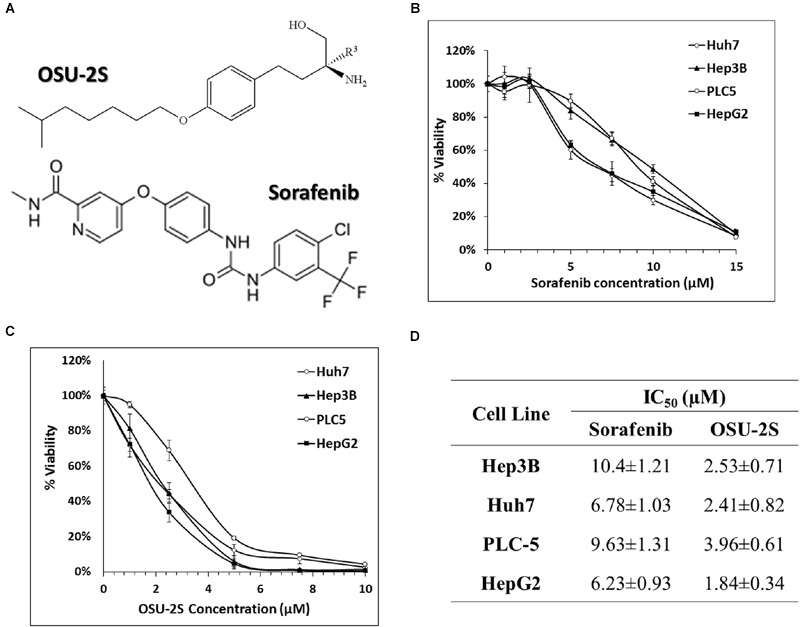
**Anti-proliferative effects of sorafenib and OSU-2S in HCC cell lines. (A)** Chemical structures of OSU-2S and sorafenib. HCC cells were treated with sorafenib **(B)** or OSU-2S **(C)** at the indicated concentrations in 10% FBS-supplemented Dulbecco’s modified Eagle’s medium (DMEM) in 96-well plates for 48 h, and cell viability was assessed by MTT assays. Points, mean; bars, SD (*n* = 6). **(D)** The IC_50_ values of sorafenib and OSU-2S in four HCC cell lines calculated from MTT assays. All data are depicted as mean ± SD (*n* = 6).

OSU-2S (**Figure 1A**) was synthesized in Dr. Chen’s lab at The Ohio State University as previously described (Omar et al., 2011). The identity and purity of OSU-2S were verified by mass spectrometry analysis and HPLC, respectively. Sorafenib (BAY 43-9006) (**Figure 1A**) was purchased from BioVision^®^ (Milpitas, CA, USA). OSU-2S and sorafenib were dissolved in DMSO and diluted in culture medium. Fetal bovine serum and MTT [3-(4,5-dimethylthiazol-2-yl)-2,5-diphenyl-2H-tetrazolium bromide] were purchased from (Sigma-Aldrich, St. Louis, MO, USA). The enhanced chemiluminescence system, Matrigel and 24-well modified Boyden chambers (8 μm pore size) were obtained from GE Healthcare Bioscience (Piscataway, NJ, USA), BD Biosciences (Bedford, MA, USA) and Corning Costar (Cambridge, MA, USA), respectively. Antibodies against various biomarkers were obtained from the following sources: PKCδ, ERKs, pERKs, from cell Signaling Technologies (Beverly, MA, USA); Poly(ADP-ribose) polymerase from Pharmingen (San Diego, CA, USA); β-actin from Sigma-Aldrich (St. Louis, MO, USA); Caspase 3 and p53 from Novus Biologicals (Littleton, CO, USA). Mammalian PKCδ shRNA expression plasmid (pKD-PKCδ-v2) and random shRNA (pKD-NegCon-v1) were purchased from Upstate (Temecula, CA, USA). Mammalian p53 shRNA expression plasmid, shp53 pLKO.1 puro was a gift from Bob Weinberg (Addgene plasmid # 19119), pLKO.1 – TRC control non-silencing plasmid was a gift from David Root (Addgene plasmid # 10879), GFP-p53 was a gift from Tyler Jacks (Addgene plasmid # 12091) and the empty vector, pEGFP-N1-FLAG was a gift from Patrick Calsou (Addgene plasmid # 60360). Other chemicals and reagents were obtained from Sigma-Aldrich unless otherwise mentioned.

### Cell Culture

The HCC cells were cultured in Dulbecco’s Modified Eagle’s medium (DMEM, Sigma-Aldrich, St. Louis, MO, USA) supplemented with 10% fetal bovine serum, 1.5 g/L sodium bicarbonate and 1% penicillin/streptomycin. PLC5 and HepG2 cells were obtained from the American Type Culture Collection (Manassas, VA, USA) while Hep3B and Huh7 cells were purchased from Sigma-Aldrich (St. Louis, MO, USA). Cell lines were maintained at 37°C in a humidified incubator containing 5% CO_2_.

### Cell Viability and Synergy Analyses

The 3-(4,5-dimethylthiazol-2-yl)-2,5-diphenyltetrazolium bro-mide (MTT) assay was used for cell viability analysis as described before (Hung et al., 2015). In summary, HCC cells were seeded in 10% FBS supplemented DMEM at 1 × 10^4^ cells per well density in 96-well flat-bottomed plates. The cells were treated with different concentrations of OSU-2S, sorafenib or their combination after 24 h of cell seeding. An equivalent volume of the used vehicle (DMSO) was used for the control treatment. After 48 h of treatment, the media were removed by aspiration and replaced by 200 μL fresh media containing 0.5 mg/mL of MTT then incubated in the CO_2_ incubator at 37°C for 2 h. At the end of the experiment, the supernatants were removed and the formed formazn crystals were dissolved in 200 μL/well DMSO. The intensity of the formed violet color was measured at 570 nm using a plate reader. Following plate reading, the data were analyzed by CalcuSyn software package version 2.1 (Biosoft, Cambridge, UK), which is based on the median effect equation to calculate the combination index (CI) of different treatments. The cell viability was expressed as percent cell vialbilty relative to the vehicle-treated control group.

### Western Blot

Lysates of OSU-2S-treated HCC cells at the indicated concentrations for 48 h were prepared for Western blotting of PARP, PKCδ, ERK1/2, pERK1/2, caspase 3, p53, and β-actin. Western blot analysis was performed as formerly reported (Arafa el et al., 2014).

### Caspase 3/7 Activity Assay

Caspase-3/7 activities in HCC cells treated with OSU-2S, sorafenib, or their combination were measured using Caspase-Glo 3/7 luminescence assay kit according to the manufacturer’s directions (Promega, Madison, WI, USA). The vehicle (DMSO) was used as negative control. In a brief, cells were seeded at 1 × 10^4^ (100 μl/well) into clear bottom, opaque wall 96-well tissue culture plates and incubated for 24 h. Cells were treated for 24 h and caspase-3/7 activities were assayed a plate luminometer.

### Invasion Assays

The assay with performed essentially as detailed before (Omar et al., 2013) with minor modifications. Hep3B cells were trypsinized and suspended in 0.5 ml of serum-free medium containing different concentrations of OSU-2S, sorafenib or their combination. The cell suspensions were seeded onto the membranes of the upper chambers of modified Boyden chambers (8 μm; Corning Costar, Cambridge, MA, USA) which were pre-coated with Matrigel. The lower chambers contained the same concentrations of the used agents in 10% FBS-containing medium. The cells were then incubated at 37°C for 24 h. After incubation, the cells remaining on the upper surface of the membranes were removed gently with cotton swabs. Cells which invaded into the lower surface of the membrane were fixed in 90% methanol and stained with 0.1% crystal violet. Stained cells were counted in at least ten 200x fields.

### Migration Assays

For the measurement of the ability of test compounds to affect cancer cell migration, the Modified Boyden chambers were used as mentioned before (Omar et al., 2009). Briefly, Hep3B in 0.5 ml of serum-free DMEM containing different concentrations of the used agents were seeded into the upper chamber membranes. The cells were incubated at 37°C for 60 min, then transferred to new wells containing the same concentrations of the used agents in 10% FBS-supplemented DMEM, and then incubated for 8 h. Non-migrated cells on the upper surface of each membrane were swabbed gently, while migrated cells into the lower side of the membrane were fixed, stained and counted as mentioned above.

### Short Hairpin (sh) RNA-Mediated PKCδ or p53 Knockdown

Hepatocellular carcinoma cells were transfected with shRNA plasmids for the knockdown of PKCδ, p53 or control vector using Lipofectamine 2000 (Life Technologies) according to the manufacturer protocol. Transfected cells with pKD-PKCδ-v2 were further subjected to stable clone isolation by 500 μg/ml geneticin (Invitrogen, Carlsbad, CA, USA) and antibiotic-resistant colonies were isolated after 2–3 weeks. The knockdown of the corresponding protein was confirmed by immunoblotting.

### Overexpression of p53

Hep3B cells were transfected with 1 μg/ml of plasmids DNA encoding GFP-p53 or the empty vector, pEGFP-N1-FLAG using Lipofectamine 2000 according to the manufacturer’s instructions and as mentioned before (Cai and Liu, 2008). The expression of p53 was confirmed by both Western blotting and fluorescence microscopy.

### Statistical Analysis

The analysis of statistical significance between different treatments was performed using one-way ANOVA followed by the Neuman–Keuls test for multiple comparisons. Differences were considered significant at *P* < 0.05. Statistical analysis was performed using SPSS for Windows (SPSS, Inc., Chicago, IL, USA).

## Results

### OSU-2S Sensitizes HCC Cells to Sorafenib-Mediated Anti-proliferative Effect

**FIGURE 2:**
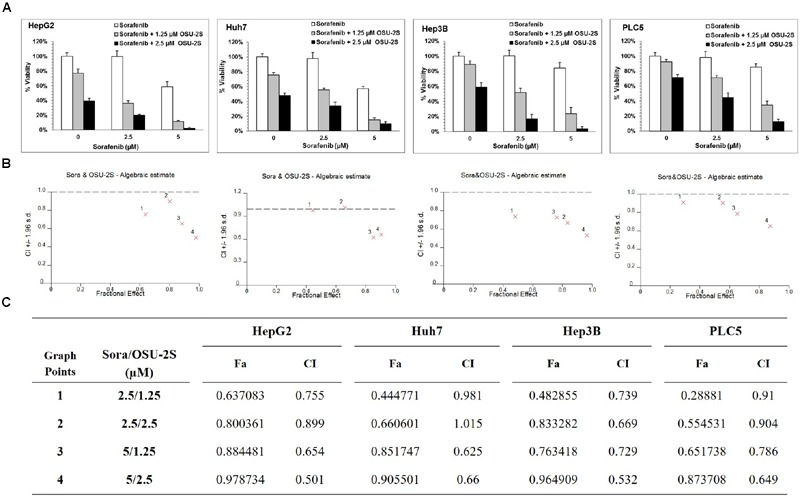
**OSU-2S/sorafenib synergistic combination. (A)** HepG2, Huh7, Hep3B, and PLC5 cell lines were treated with sorafenib or OSU-2S at the indicated concentrations in 10% FBS-DMEM plates for 48 h, and cell viability was assessed by MTT assays. Columns, mean; bars, SD (*n* = 6). **(B)** OSU-2S/sorafenib combination algebraic estimate calculated by Calcusyn software. **(C)** A table showing the fraction affected (Fa) and OSU-2S/sorafenib combination indices (CI) at the indicated dose levels in four different HCC cell lines.

The ability of OSU-2S and sorafenib as a single agent to inhibit the cell viability of HCC cell lines has been reported before (Ng and Chen, 2006; Omar et al., 2011). In order to select a suitable range of drug concentrations for combination experiments, the effect of both sorafenib and OSU-2S on the cell viability of four different HCC cell lines was initially investigated using MTT assay. The dose response curve of sorafenib or OSU-2S was assessed relative to vehicle control treatment (**Figures 1B,C**). The half maximum inhibitory concentration (IC_50_) for OSU-2S was in the range of 1.8–3.9 μM with the HepG2 cells being the most sensitive and the PLC-5 cells being the most resistant. For sorafenib, the IC_50_ was in the range of 6.2–10.4 μM with HepG2 cells being the most sensitive and Hep3B cells being the most resistant (**Figure 1D**). Sorafenib-mediated anti-proliferative effect was significantly enhanced upon the combination with OSU-2S. Combination indexes (CI) were calculated using Calcusyn software for each dose combination. Values of CI <1 indicate synergy, =1 indicate additive effect and >1 indicate antagonism. Synergistic effects were observed in the four used HCC cell lines with different degrees. For example, in HepG2, Hep3B, and PLC-5 cells, almost all the selected dose levels showed synergistic effect. While in Huh7 cells, 2 out of 4 of the combination concentrations showed additive effects (**Figure 2**).

### OSU-2S Sensitizes HCC Cells to Sorafenib-Mediated Anti-proliferative Effect

**FIGURE 3:**
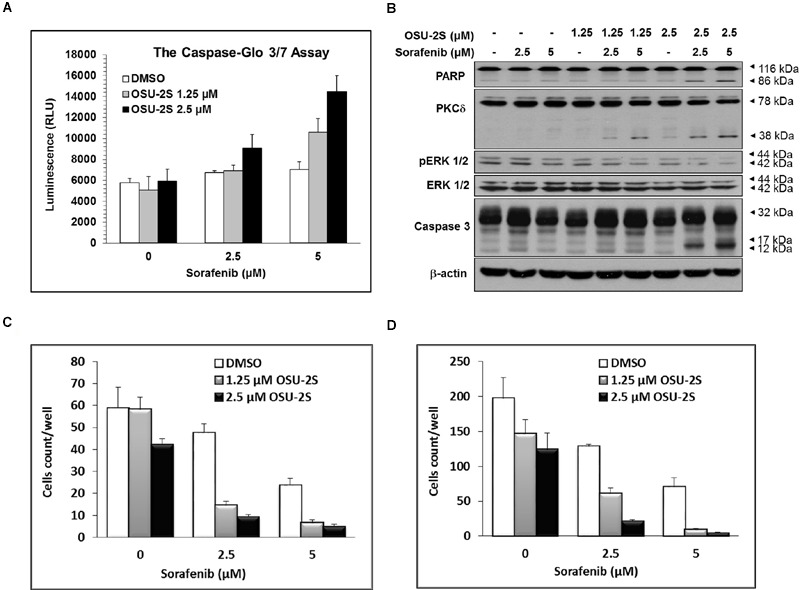
**OSU-2S sensitizes HCC Cells to sorafenib-mediated anti-proliferative effect. (A)** Caspase 3/7 activities were measured using the Caspase-Glo Assay Kit and **(B)** Western blot analysis of the expression levels of PARP, PKCδ, pERK1/2, ERK1/2 and caspase-3 in Hep3B cells after the indicated treatment for 48 h in 10% FBS-supplemented DMEM. All data are depicted as ±SD (*n* = 6). Effects of sorafenib/OSU-2S combination on Hep3B cells **(C)** invasion and **(D)** migration. Columns, mean; bars, SD (*n* = 10).

The ability of sorafenib/OSU-2S combination to elicit apoptotic cell death compared to single drug treatment was initially tested using caspases 3/7 activity assay. For this experiment, Hep3B cells were selected since it was the most resistant to sorafenib as a single agent. Results showed that increasing doses of OSU-2S dramatically increased sorafenib-induced activation of caspases 3/7 (1.5- to 3-fold increase) especially at 2.5 μM dose level of OSU-2S (**Figure 3A**). These results were confirmed by Western blotting of two hallmarks of apoptosis, caspase 3 and its downstream target protein, PARP. The results showed a significant increase in caspase 3 activation through cleavage with a parallel increase in PARP cleavage (**Figure 3B**).

In addition, the possible modulatory effects of OSU-2S on the reported anticancer mechanism of sorafenib were investigated by Western blotting of ERK1/2 phosphorylation, which is considered as a major target of sorafenib (Adnane et al., 2006; Manov et al., 2011). Results showed that OSU-2S increased the inhibitory effect of sorafenib on ERK1/2 phosphorylation (**Figure 3B**). At the same time, sorafenib/OSU-2S combination displayed a significant increase in PKCδ activation as a reported mechanism for OSU-2S and another biomarker for apoptosis (Omar et al., 2011) (**Figure 3B**).

### Sorafenib/OSU-2S Combination Inhibits *In vitro* Cell Migration/Invasion of HCCs

The ability of OSU-2S to synergize the effect of sorafenib on endothelial cell migration/invasion was analyzed by modified Boyden’s chamber assay. Only cancer cells with high migratory ability can pass through the Boyden’s chamber membrane of 8 μm pore. Sorafenib effectively inhibited the ability of HCC cells to invade Matrigel-coated membranes in a dose-dependent manner (**Figure 3C**). In addition, sorafenib inhibited the migratory ability of HCC cells through the porous inserts (**Figure 3D**). The sorafenib/OSU-2S combination showed a significant synergy in sorafenib-mediated inhibition of both migration and invasion.

### Sorafenib/OSU-2S Combination Synergy Is, in Part, Mediated through PKCδ Activation

**FIGURE 4:**
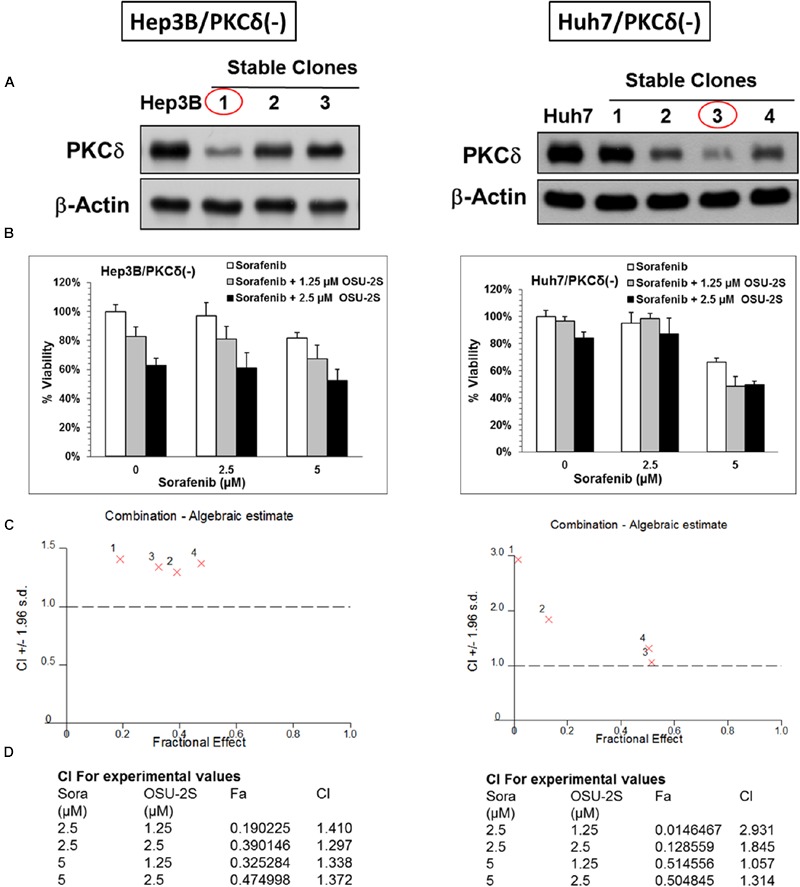
**PKCδ Knockdown abolishes the synergy between sorafenib and OSU-2S.** The effect of PKCδ Knockdown on the anti-proliferative activity of sorafenib/OSU-2S combination. **(A)** Western blot analysis of the differential expression levels of PKCδ in untransfected (*left*) Hep3B and (*right*) Huh7 cells versus different corresponding stable clones. Effect of sorafenib/OSU-2S combination on **(B)** the viabilities of (*left*) Hep3B and (*right*) Huh7 cells stable clones with PKCδ Knockdown (#1 and #3), **(C)** The combination algebraic-estimate and **(D)** the combination indices. Columns, mean; bars, SD (*n* = 6).

PKCδ, a pro-apoptotic kinase, is involved in caspase-3-dependent apoptotic pathway in HCC (Reyland, 2007; Hung et al., 2008). The role of PKCδ activation as a possible mechanism for the synergy between sorafenib and OSU-2S was studied through the knockdown of PKCδ of from Hep3B and Huh7 cell lines. The knockdown of PKCδ protein was initially confirmed by Western blotting and the stable clones with the lowest expression levels of PKCδ was used for the following MTT analysis (**Figure 4A**). PKCδ knockdown caused a significant increase in the resistance of both Hep3B and Huh7 to sorafenib and OSU-2S with about 2- to 3.5-fold increase in the IC_50_ (**Figures 4B,C**). In addition, PKCδ knockdown completely eliminated the synergy between sorafenib and OSU-2S in their combination as indicated by all CI values over 1 (**Figure 4D**). These results suggested the activation of PKCδ as a putative mechanism for synergistic sorafenib/OSU-2S combination.

### The Role of p53 in the Sensitivity of HCC Cells to Both Sorafenib and OSU-2S

**FIGURE 5:**
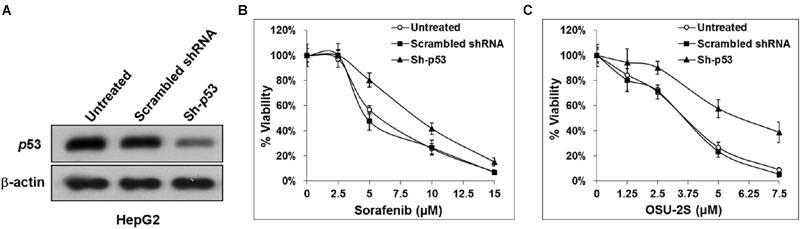
**Knockdown of p53 in HepG2 cells increases the resistance to both sorafenib and OSU-2S. (A)** Western blot analysis of the differential expression levels of p53 in untransfected HepG2 cells versus HepG2 cells transfected by scrambled shRNA or shRNA against p53. Effect of p53 knockdown on the sensitivity of HepG2 cells to **(B)** sorafenib or **(C)** OSU-2S-mediated inhibition of cell viability measured by MTT assay. Points, mean; bars, SD (*n* = 6).

**FIGURE 6:**
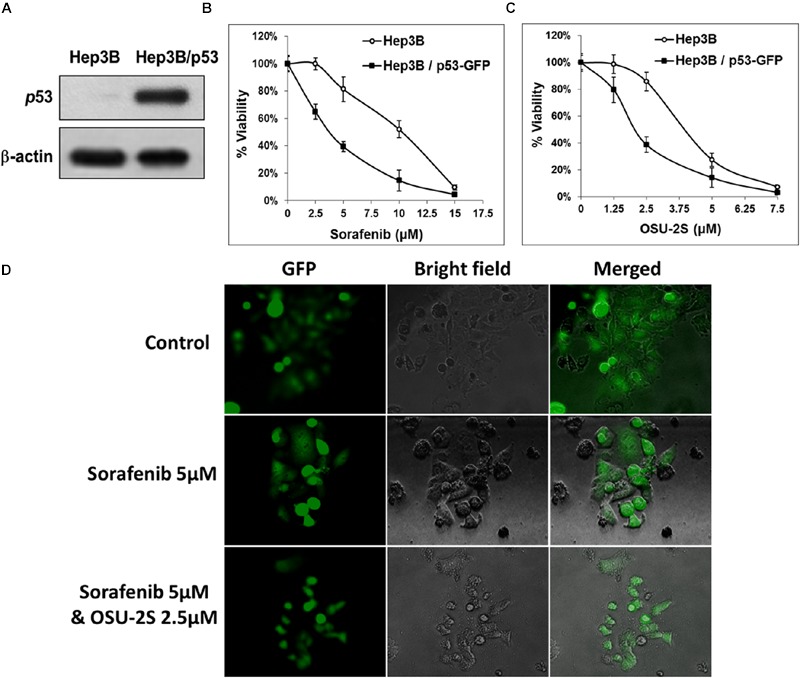
**The over-expression of p53 in Hep3B cells restores the sensitivity to both sorafenib and OSU-2S. (A)** Western blot analysis of the differential expression levels of p53 in untransfected Hep3B cells versus Hep3B cells over-expressing p53. Effect of p53 over-expression on the sensitivity of Hep3B cells to **(B)** sorafenib or **(C)** OSU-2S-mediated inhibition of cell viability measured by MTT assay. Points, mean; bars, SD (*n* = 6). **(D)** Fluorescence and direct light microscopy of Hep3B cells over-expressing p53 on the indicated treatments.

The used HCC cell lines showed differential sensitivity to both sorafenib and OSU-2S. HepG2 was the most sensitive to both drugs. Among the four used HCC cell lines in this study, only HepG2 cells have wild type functional p53 while the others lack functional p53 due to deletion or mutation (Lee et al., 2002). Based on this observation and based on the central role of p53 in apoptotic cell death, the lack of functional p53 in Hep3B postulated as a mechanism of resistance. To elucidate the possible role of the presence of functional p53 in HCC cells in the sensitivity or the resistance of sorafenib and OSU-2S, p53 knockdown was performed in HepG2 cells followed by MTT assay. Results showed that the p53 knockdown caused a significant increase in the resistance of HepG2 cells toward both sorafenib and OSU-2S with IC_50_ values of 9.9 and 6.1 μM, respectively, which is almost doubling of the IC_50_ values (**Figures 5A–C**). In addition, p53 over-expression was performed in Hep3B cells, which were the most resistant to sorafenib followed the MTT assay. The results showed that restoring the activity of p53 caused the Hep3B cells to be much more sensitive to both sorafenib and OSU-2S with IC_50_ values of 3.2 and 1.9 μM, respectively (**Figures 6A–C**). Furthermore, the anti-proliferative activity of sorafenib/OSU-2S combination was significantly increased upon p53 restoration by the overexpression as indicated by the progressive morphological changes from flat to round in Hep3B-p53-GFP which are characteristic of apoptosis (**Figure 6D**). These results suggested a significant role of p53 in the sensitivity of HCC cells to both sorafenib and OSU-2S as single agents or in combination.

## Discussion

The low efficacy of sorafenib in clinical practice due to *de novo* resistance or dose reductions to avoid the full dose adverse effects raised the current need for combination therapies with sorafenib (Hikita et al., 2010; Xie et al., 2012; Federico et al., 2015). The present study provides an evidence on the synergistic combination of sorafenib with the novel anticancer agent, OSU-2S and sheds light on their mechanism of synergy and translational potential into clinical application. In a previous study, we have addressed the anti-proliferative mechanism of OSU-2S in HCC, both *in vitro* and *in vivo*, through the activation of PKCδ signaling pathways and the subsequent induction of caspase-dependent apoptosis (Omar et al., 2011).

The design of sorafenib/OSU-2S combination was based on the ability of sorafenib to induce p53 family-dependent apoptosis in HCC and the lack of functional p53 in most HCC cells which could be a possible mechanism for sorafenib resistance (Brost et al., 2013; Wei et al., 2015). Then, it was rational to combine sorafenib with OSU-2S to overcome sorafenib resistance through the ability of OSU-2S to activate PKCδ signaling which, in turn, stimulates p53-dependent and -independent apoptotic cell death pathways (Hew et al., 2011).

As single agents, OSU-2S or sorafenib exhibited moderate anticancer activities against the used HCC cell lines while their combinations were potentially synergistic. The synergy was supported by the observed increase in apoptotic cell death hallmarks like caspase 3/7 activation and PARP cleavage. From a mechanistic perspective, OSU-2S displays a unique ability to activate PKCδ and caspase-dependent apoptosis (Omar et al., 2011). The hypothesis that OSU-2S augmented the anti-proliferative effect of sorafenib in HCC cell lines, in part, through the activation of PKCδ was supported by the absence of synergy upon PKCδ knockdown.

PKCδ has a contrasting role in regulating apoptotic cell death, either proapoptotic or antiapoptotic, in different cell systems (Brodie and Blumberg, 2003; Basu and Pal, 2010). Previously, Hung et al. (2008) demonstrated that the activation of PKCδ in HCC cells through proteolytic cleavage elicited apoptotic cell death rather than survival. Similarly, the activation of PKCδ through proteolytic cleavage followed by nuclear translocation or allosteric activation caused significant inhibition of proliferation and apoptosis different cancer cells (Fujii et al., 2000; DeVries et al., 2002). On the other hand, PKCδ inhibition was reported as a key player for sensitizing TRAIL-resistant human fibrosarcoma (Hayashi et al., 2014). Other studies indicated that activation of PKC with phorbol-12-myristate-13-acetate (PMA), blocks TRAIL, and TNF-α induced apoptosis (Sarker et al., 2001; Harper et al., 2003). The discrepancies in the consequence of PKCδ activation whether enhancement or suppression of apoptosis appears to depend on the initiating signal and the type of cancer cells (Garg et al., 2014).

It is worth noting that PKCδ has also been shown to suppress cell migration, and its absence could contribute to both cell survival and metastasis in human cancers (Jackson et al., 2005). In the current study, the activation of PKCδ by OSU-2S caused a significant inhibition in HCC cell invasion and migration. In similar reports, the overexpression of PKCδ inhibited breast cancer cell migration (Jackson et al., 2005). On the contrary, PKCδ activity was required in integrin-mediated metastatic melanoma invasion and EGFR-induced migration in fibroblasts (Iwabu et al., 2004; Putnam et al., 2009). Also, HIF-2α promoted PKCδ-mediated migration in HCC through enhanced phosphorylation (Cao et al., 2016). Since there is a considerable heterogeneity within tumor cells and due to the involvement of several signaling pathways in the process of migration/invasion, the events following PKCδ activation could vary depending on the cell type and the used stimulus (Basu and Pal, 2010).

*In vitro* studies showed that PKCδ enhances cancer cell apoptosis by antagonizing ERK phosphorylation (Li et al., 2012). The potentiated effect of the combination on ERK phosphorylation can be explained based on the reported ability of PKCδ to inhibit ERK phosphorylation, which is similar to the major anticancer mechanism of sorafenib (Li et al., 2012). Similarly, PKCδ inhibited hepatocyte growth factor (HGF)-induced phosphorylation of ERK (Hu et al., 2013). In addition, PKCδ activity was required to activate the pro-apoptotic ERK signaling during B cell development (Limnander et al., 2011). Conversely, PKCδ activation through phosphorylation induced a sustained activation of ERK in response to etoposide-induced apoptosis in glioma cells (Lomonaco et al., 2008). The difference in response to PKCδ activation among MAPK family members could be explained based on the way of PKCδ activation being through proteolytic cleavage or allosteric and the nature of cells.

In this study, we demonstrated that p53 status in HCC cells predicts their sensitivity toward both sorafenib and OSU-2S. The tumor-suppressor protein p53 is a master regulator of apoptosis, in response to cellular stress (Farnebo et al., 2010). Since the tumor-suppressing effects of PKCδ are mediated at least in part through activating p53 transcription (Abbas et al., 2004), the existence of wild type p53 in HepG2 cells could partially explain their relatively higher sensitivity to both drugs than the other HCC cell lines which lack functional p53. The role of p53 in the differential sensitivity to both sorafenib and OSU-2S was confirmed by abrogating the sensitivity of HepG2 cell by p53 knockdown and rendering Hep3B cells much more sensitive by p53 overexpression. This observation can be also supported by the ability of sorafenib to up-regulate p53 expression and to induce p53 family-dependent apoptosis in HCC cells (Fernando et al., 2012; Wei et al., 2015).

## Conclusion

OSU-2S could effectively augment the anti-proliferative effect of sorafenib in HCC cell lines, in part, through the activation of PKCδ. The p53 status in HCC cells predicts their sensitivity toward both sorafenib and OSU-2S. The current study underscores evidence about the translational potential of OSU-2S/sorafenib combination and encourages future *in vivo* safety studies to allow the extrapolation into the clinical setting as a therapeutically relevant approach for HCC patients.

## Author Contributions

Conceived and designed the experiments: HO, MT, J-HH, and TA-T. Performed the experiments: HO, MT, and J-HH. Analyzed the data: HO, MT, J-HH, and TA-T. Contributed reagents/materials/analysis tools: HO, MT, J-HH, and TA-T. Wrote and revised the manuscript: HO, MT, J-HH, and TA-T.

## Conflict of Interest Statement

The authors declare that the research was conducted in the absence of any commercial or financial relationships that could be construed as a potential conflict of interest.
